# Epidemiology of patients with suicidal intent in the emergency department at the University Hospital of Salamanca

**DOI:** 10.1192/j.eurpsy.2025.2345

**Published:** 2025-08-26

**Authors:** E. D. Alvarez, P. A. Olivera, M. L. Argudo, I. M. P. Navarro, C. G. Cerdan, C. M. Cossio, R. K. G. Bolaños, R. M. B. Rey, C. P. Rodriguez, L. M. D.-P. Barros, P. S. Aranda, I. R. Blanco, C. M. Lorenzo, A. S. Chillon, R. A. S. Limno, C. E. B. Mercado

**Affiliations:** 1 CAUSA, Salamanca, Spain

## Abstract

**Introduction:**

Suicide represents a serious public health problem. Suicidal gestures make up a significant proportion of the psychiatric emergencies treated in Emergency Services.

**Objectives:**

The study aimed to identify which of the total emergency visits were related to suicidal ideation or suicide attempts, and to analyze the epidemiology of these patients in terms of age and gender.

**Methods:**

A retrospective, observational study was conducted by collecting and analyzing data from July 2024 in the Emergency Department of the Clinical Hospital of Salamanca.

**Results:**

Out of a total of 201 patients treated by on-call psychiatry in the Emergency Department, 64 patients (31.8%) presented suicidal ideation or suicide attempts. Of these, 43 were women (67%) and 21 were men (33%). By age group, among the women, 5 were under 18 years old, 11 were between 18 and 35 years old, 16 were between 36 and 55 years old, and 11 were over 55 years old. Among the men, 1 was under 18 years old, 7 were between 18 and 35 years old, 8 were between 36 and 55 years old, and 5 were over 55 years old.

**Conclusions:**

Regarding the profile of patients with suicidal behavior, there is a higher presence of women with suicidal tendencies, as reported in the literature and other studies. No clear relationship was observed between age ranges and suicide attempts. It is important to note that the sample size is not statistically significant to determine representativeness.

**Disclosure of Interest:**

None Declared

